# Metagenomic Exploration of Viruses throughout the Indian Ocean

**DOI:** 10.1371/journal.pone.0042047

**Published:** 2012-10-17

**Authors:** Shannon J. Williamson, Lisa Zeigler Allen, Hernan A. Lorenzi, Douglas W. Fadrosh, Daniel Brami, Mathangi Thiagarajan, John P. McCrow, Andrey Tovchigrechko, Shibu Yooseph, J. Craig Venter

**Affiliations:** 1 Microbial and Environmental Genomics, J. Craig Venter Institute, San Diego, California, United States of America; 2 Informatics, J. Craig Venter Institute, Rockville, Maryland, United States of America; 3 Informatics, J. Craig Venter Institute, San Diego, California, United States of America; 4 University of California San Diego, Scripps Institution of Oceanography, La Jolla, California, United States of America; Argonne National Laboratory, United States of America

## Abstract

The characterization of global marine microbial taxonomic and functional diversity is a primary goal of the Global Ocean Sampling Expedition. As part of this study, 19 water samples were collected aboard the *Sorcerer II* sailing vessel from the southern Indian Ocean in an effort to more thoroughly understand the lifestyle strategies of the microbial inhabitants of this ultra-oligotrophic region. No investigations of whole virioplankton assemblages have been conducted on waters collected from the Indian Ocean or across multiple size fractions thus far. Therefore, the goals of this study were to examine the effect of size fractionation on viral consortia structure and function and understand the diversity and functional potential of the Indian Ocean virome. Five samples were selected for comprehensive metagenomic exploration; and sequencing was performed on the microbes captured on 3.0-, 0.8- and 0.1 µm membrane filters as well as the viral fraction (<0.1 µm). Phylogenetic approaches were also used to identify predicted proteins of viral origin in the larger fractions of data from all Indian Ocean samples, which were included in subsequent metagenomic analyses. Taxonomic profiling of viral sequences suggested that size fractionation of marine microbial communities enriches for specific groups of viruses within the different size classes and functional characterization further substantiated this observation. Functional analyses also revealed a relative enrichment for metabolic proteins of viral origin that potentially reflect the physiological condition of host cells in the Indian Ocean including those involved in nitrogen metabolism and oxidative phosphorylation. A novel classification method, MGTAXA, was used to assess virus-host relationships in the Indian Ocean by predicting the taxonomy of putative host genera, with *Prochlorococcus*, *Acanthochlois* and members of the SAR86 cluster comprising the most abundant predictions. This is the first study to holistically explore virioplankton dynamics across multiple size classes and provides unprecedented insight into virus diversity, metabolic potential and virus-host interactions.

## Introduction

Viruses (predominantly bacteriophages) are the most abundant biological components of marine ecosystems, generally outnumbering their microbial hosts by an order of magnitude [1]. Viruses are significant agents of microbial mortality, influencing diversity, and play an integral role in marine ecosystem processes such as nutrient transformation and cycling [2]–[4]. Through interactions with microbes, viruses also influence the global flow of genes affecting host cell phenotype, niche adaptation and evolution [5]. Metagenomic investigations of viruses collected from varied marine ecosystems have provided insight into local and global viral diversity [6]–[8], genotypic distribution [9], [10], functional potential [11]–[15] and replication strategy [16], [17]. The majority of these metagenomic studies have been conducted on the viral fraction of marine samples, where a pre-filtration step (generally ranging from 0.22- to 0.45 µm) was used to physically separate viral particles from cellular organisms. Alternatively, metagenomic investigations have been conducted on virus-like sequences contained within the cellular fraction of marine samples, including surface water samples collected during the first reported phase of the Global Ocean Sampling (GOS) Expedition (termed Phase I) [12], and a depth profile collected from the HOT station ALOHA [18]. In these instances, viral sequences were identified based on their homology to known viruses. Each of these strategies has its limitations. Examinations of viruses within the cellular fraction of metagenomic data alone inevitably results in an underestimation of viral sequences due to its dependency on similarity to previously sequenced viruses and are constrained to only those viruses (or their nucleic acids) that were physically captured. Similarly, evaluation of only the viral fraction can result in a less than comprehensive picture of viral diversity and functional capacity since it excludes viruses removed through pre-filtration.

To date, no investigations of whole virioplankton assemblages have been conducted on waters collected from the Indian Ocean, however, targeted studies of cyanophage [19] and a virus infecting a heterotrophic flagellate [20] have been reported. In order to gain a more thorough understanding of the GOS Indian Ocean (GOS-IO) viromes and to better appreciate the implications of size fractionation, both the cellular and viral fractions were examined using metagenomic approaches that target dsDNA viral sequences. Virus-host associations were also evaluated using a novel classification method that predicts the taxonomy of putative microbial hosts using assembled viral metagenomic data based on polynucleotide compositional signatures described by Interpolated Context Models (ICMs) adopted from Phymm [21]. This study represents the first comparative analysis of viruses across multiple size classes from marine water samples.

## Results and Discussion

### Dataset characteristics and identification of viral sequences from filter fractions

**Figure 1:**
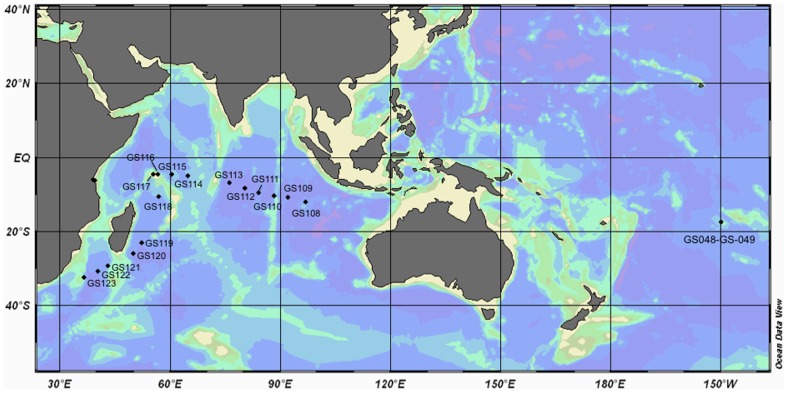
Map of Indian Ocean indicating where samples for metagenomic analysis were collected.

Surface water samples (∼400 L) were collected from 17 sites from the tropical Indian Ocean between August and October 2005 aboard the S/V Sorcerer II. Two additional sites were sampled off the island of Zanzibar, Tanzania using alternate vessels (**Figure 1** **; Table S1**). The microbial community was pre-filtered using 20 µm mesh Nytex net and then size fractionated by serial filtration through 3.0-, 0.8-, and 0.1 µm membrane filters. The viral fraction of water samples (i.e. <0.1 µm) was concentrated and purified as described previously [22] (see Materials and Methods for details). DNA was extracted from microbial cells and viral particles as previously described [22], [23] and sequenced using a combination of Sanger and pyrosequencing technologies.

While the microbes retained within the 0.1–0.8 µm size fraction were sequenced for all samples, five independent samples were selected for comprehensive metagenomic exploration with sequencing performed on all microbial size fractions 20-0.1 µm, including the viral fraction (<0.1 µm) (**Table 1** **; Table S2**). The five samples that were comprehensively sequenced were selected based on sufficient quantity and quality of DNA across all size fractions. A total of approximately 228K Sanger and 2.2M 454 Titanium sequence reads were produced from the five viral libraries, resulting in a combined 2.7M predicted ORFs (see Materials and Methods for details on the ORF predication pipeline). The Sanger and 454 datasets had average read lengths of 989 bp and 360 bp, respectively. Despite rigorous purification of the viral concentrates (VCs) prior to metagenomic library construction, it became apparent during sequence analysis that one library, GSIOVIR110, contained non-trivial amounts of cellular contamination similar to sequences found in the larger filter data (0.1–20 µm). Therefore, this sample was not included in subsequent analyses with the exception of co-assembly with data from all size fractions.

**Table 1:** Sanger and 454 pyrosequencing statistics for Indian Ocean viral metagenomes.

Sample	# Sequences	Avg. Read Length (bp)	%GC	# Predicted Proteins	% Phylogenetic Trees	% Unknown
***Sanger***						
GSIOVIR108	44,504	990	39.1	70,563	14.42	12.5
GSIOVIR110*	47,572	977	35.8	73,947	n/a	8.8
GSIOVIR112	46,443	1,003	38.2	74,721	26.70	10.7
GSIOVIR117	43,530	992	38.8	74,679	21.56	12.4
GSIOVIR122	46,584	983	39.3	71,007	23.69	11.3
**Total**	**228,633**	**989**	**38.2**	**364,917**	**Avg. = 21.59**	**Avg. = 11.1**
***454***						
GSIOVIR108	318,094	389	42	254,792	14.12	15.2
GSIOVIR110*	331,693	329	43	231,953	n/a	12.7
GSIOVIR112	492,396	358	42	344,886	17.91	11.3
GSIOVIR117	710,101	378	41	574,756	14.12	12
GSIOVIR122	337,266	347	45	226,097	22.41	12.5
**Total**	**2,189,550**	**360**	**40.4**	**2,362,318**	**Avg. = 17.14**	**Avg. = 11.9**

* Sample not used in taxonomic and functional analyses due to cellular contamination. n/a denote where the data was not used in study. Percent of phylogenetic trees denotes the amount of sequences (ORFs) that were taxonomically classified via APIS.

The identification of viral sequences within the metagenomic data from larger size fractions was accomplished using a phylogenomic approach, the Automated Phylogenetic Inference System (APIS), that was designed for annotation of genomic [24] and metagenomic [25] datasets. APIS taxonomically and functionally classifies each sequence inferred from neighbor-joining phylogenetic trees (see Materials and Methods for more details); unlike previous reports using homology-based methods [12], [18]. This analysis resulted in the identification of 102,790 predicted proteins of viral origin within the larger size fractions (0.1–20 µm) (**Table S2**), representing 2.8% of the total predicted proteins from the Indian Ocean microbial dataset (∼3.6M total predicted proteins). This estimate is almost identical to a previous study which found that viral sequences accounted for ∼3% of GOS Phase I microbial data collected between Nova Scotia and French Polynesia [12].

### Viral genotypic diversity

Viral genotypic diversity was estimated at the Indian Ocean sampling locations using the Phage Communities from Contig Spectrum (PHACCS) tool [26]. Richness, evenness and diversity estimates were obtained for each viral library using the complete set of reads generated upon sequencing. Comparisons were performed across the following categories based on the contig spectrum produced from Newbler-based assemblies of: 1) fragmented Sanger data (i.e. unmated), 2) paired-end Sanger data, 3) all 454 data, and 4) sub-sampled 454 data (**Table 2** **,** **Table 3** **, and Table S3**). Estimates of genotype richness varied widely between the Sanger and most of the 454 categories, but the relative ranking was conserved. On average, the most diverse site among all assemblies tested was GSIOVIR117, which also had the largest number of genotypes. The least diverse site, on average, was GSIOVIR122, which also had the lowest number of genotypes (**Table 2** **and** **Table 3**). Evenness estimates were similar across categories with a slight increase in assemblies that utilized all of the 454 data per library rather than sub-samples (**Table 2**). Richness and evenness estimates, along with the most abundant genotype percentages, suggest that all sites harbored diverse viral populations with most of the diversity residing in the “long tail” of the genotype distribution.

**Table 2:** Estimated diversity of Indian Ocean virioplankton assemblages using PHACCS.

Sample	Richness (# genotypes)	Evenness	SW Index (nats)	Most abundant genotype (%)
***Sanger_Frag***				
GSIOVIR108	3,327	0.85	6.86	5.62
GSIOVIR112	10,592	0.85	7.84	4.32
GSIOVIR117	22,040	0.88	8.83	2.47
GSIOVIR122	554	0.89	5.59	6.63
***Sanger_PE***				
GSIOVIR108	1,309	0.86	6.17	6.36
GSIOVIR112	10,560	0.85	7.83	4.34
GSIOVIR117	17,069	0.9	8.73	2.23
GSIOVIR122	388	0.89	5.32	6.96
***454_All***				
GSIOVIR108	1,237	0.93	6.6	3.14
GSIOVIR112	4,337	0.91	7.59	2.81
GSIOVIR117	4,850	0.92	7.84	2.09
GSIOVIR122	1,387	0.91	6.61	3.65
***454_Sub***				
GSIOVIR108	405±79	0.89±0.005	5.34±0.15	6.95±0.22
GSIOVIR112	26,703±844	0.84±0.003	8.55±0.05	3.79±0.13
GSIOVIR117	211,276±8,728	0.80±0.003	8.06±0.40	5.37±0.38
GSIOVIR122	559±98	0.88±0.005	5.55±0.14	6.97±0.17

Frag: fragment; PE: paired end; Sub: random subsamples; SW: Shannon-Weiner.

**Table 3:** Estimated diversity using varied genome sizes (kb) via PHACCS.

Richness	100 kb			80 kb			50 kb (§)			25 kb			15 kb		
**GS108viral**	212	+/−	43	261	+/−	52	405	+/−	79	784	+/−	146	1,283	+/−	233
**GS112viral**	340,000	+/−	113,137	38,437	+/−	2,966	26,703	+/−	844	300,000	+/−	282,843	73,000	+/−	10,850
**GS117viral**	109,914	+/−	312,933	15,521	+/−	23,108	211,276	+/−	8,728	119,072	+/−	309,835	26,434	+/−	19,148
**GS122viral**	120,226	+/−	315,429	367	+/−	67	559	+/−	98	1,062	+/−	178	1,720	+/−	281
**Evenness**															
**GS108viral**	0.88	+/−	0.01	0.89	+/−	0.01	0.89	+/−	0.01	0.9	+/−	0.01	0.91	+/−	0.01
**GS112viral**	0.72	+/−	0.01	0.79	+/−	0	0.84	+/−	0	0.78	+/−	0.02	0.86	+/−	0
**GS117viral**	0.79	+/−	0.04	0.82	+/−	0.02	0.8	+/−	0	0.83	+/−	0.03	0.86	+/−	0.01
**GS122viral**	0.8	+/−	0.15	0.87	+/−	0.01	0.88	+/−	0.01	0.89	+/−	0	0.9	+/−	0
**SW Index (nats)**															
**GS108viral**	4.72	+/−	0.14	4.92	+/−	0.14	5.34	+/−	0.15	6	+/−	0.13	6.48	+/−	0.13
**GS112viral**	9.07	+/−	0.21	8.36	+/−	0.04	8.55	+/−	0.01	9.68	+/−	0.29	9.57	+/−	0.11
**GS117viral**	7.42	+/−	0.91	7.41	+/−	0.64	8.06	+/−	0.4	8.36	+/−	0.85	8.58	+/−	0.53
**GS122viral**	5.26	+/−	0.79	5.13	+/−	0.15	5.55	+/−	0.14	6.18	+/−	0.14	6.66	+/−	0.14
**Most abundant genotype (%)**															
**GS108viral**	9.32	+/−	0.15	4.05	+/−	0.03	5.05	+/−	0.04	6.95	+/−	0.22	8.44	+/−	0.12
**GS112viral**	6.24	+/−	0.01	2.64	+/−	0.01	4.22	+/−	0.28	3.79	+/−	0.13	5.19	+/−	0.01
**GS117viral**	6.3	+/−	0.36	3.27	+/−	0.38	4.22	+/−	0.21	5.37	+/−	0.38	5.82	+/−	0.49
**GS122viral**	10.54	+/−	2.34	4.23	+/−	0.08	5.22	+/−	0.1	6.97	+/−	0.17	8.51	+/−	0.24

(§) indicates the genome size that was used in Table 2.

To account for the possibility of varying genome size, diversity estimates were also obtained on the sub-sampled 454 datasets using a range of average viral genome sizes (**Table 3**). For these analyses, sample richness varied greatly depending on the average genome size used. The greater richness estimates (e.g. >100k genotypes) could reflect a limit in the PHACCS algorithm and likely do not reflect a relevant genome size estimation. It has been reported that the average genome size of marine viruses is typically between 50–80 kb [27], [28]. Our data suggests the same based on estimates of richness, with an interesting change in GSIOVIR117, where the richness estimates were more in line with the Sanger estimates when using an estimated genome size of 80 kb (**Table 3**). Lastly, as mentioned above, the relative ranking of samples with respect to diversity estimates was conserved and corresponded to the total number of predicted peptides per site. However, previous reports have suggested that there is no link between sequencing depth and the number of genotypes [26]. Tools for genome size estimations of viruses are available; however depend heavily on reference databases. Based on phylogenetic classification of ORFs from sequence reads, ∼20% of the GOS-IO data had significant similarity to a known sequence and therefore was classified (**Table 1**). Thus, we feel current approaches are not directly applicable as yet to these data.

To the best of our knowledge, this is the first study to compare PHACCS-based estimations of viral diversity using sequence information produced from two different platforms (Sanger and 454), yet assembled using the same methods. The number of predicted viral genotypes (richness) in a sample was the most variable with respect to the type of data produced (Sanger mated and unmated; 454 all and sub-samples). Sub-sampling of datasets is recommended to reduce coverage and improve the quality of PHACCS estimations.

This comparison demonstrated that estimations of viral diversity using PHACCS, particularly the number of predicted genotypes, may be influenced by the nature of viral data and their subsequent assembly; yet most estimates fell within the range of previously published reports [6]–[8]. Furthermore, measurements of viral consortia diversity were underestimated since viruses retained in the larger size classes were excluded from the analysis, as they were not included in the assemblies. Subsequent taxonomic and functional assessments (discussed below) suggest that viral sequences found in the microbial (cellular) fraction are distinct compared to those generated from the viral-size fractions.

### Taxonomic binning of Indian Ocean virome

Sequences originating from discrete viral fractions (VFs) and the larger fractions (LFs) were taxonomically characterized using two approaches. One approach was based on a BLASTP comparison against the NCBI non-redundant (nr) protein database, which does not include predicted protein sequences from viral metagenomes. The other was a phylogenomic approach using APIS. Examination of metagenomic data using APIS resulted in ∼20% of sequences from the VFs producing phylogentic trees, and thus taxonomic classification (**Table 1**). The low level of APIS classification was due to the stringency of the method (see Materials and Methods for details). The majority of predicted protein sequences from the VF were characterized as cellular using both the homology (68%) and phylogenetic-based approaches (43%) (**Table 4**), despite evidence that cellular contamination was not present in the viral samples (i.e. no amplification of the 16S rRNA gene during library construction; see Materials and Methods for details). A larger proportion of sequences were classified as Virus at the kingdom level through APIS (56%) than by homology comparison to the non-redundant (nr) database (32%), due to the inclusion of viral metagenomic data of diverse origin within the APIS database. As expected due to the sample processing methodology employed, viral sequences originating from the VF as well as the LF were most similar to dsDNA viruses; however, the most abundant taxonomic group varied.

**Table 4:** Taxonomic characterization of Indian Ocean virioplankton assemblages.

Taxa	% LF APIS	% VF APIS	% VF BLAST
***“Kingdom*** a ***”***			
Cellular	NA	42.6	68
Virus	2.8	55.6	32
Other	NA	NA	0.1
Mixed	NA	1.8	NA
***“Virus*** b ***”***			
Metagenomic dsDNA	29.2	80.3	NA
dsDNA viruses	39.9	17.2	97.34
ssDNA viruses	0	0	0.08
ssRNA viruses	0	0	0.07
dsRNA viruses	0	0	0.01
Retro-transcribing viruses	0	0	0.02
Unclassifed phage	NA	NA	2.45
Unclassifed archaeal phage	0	0	0.05
Unclassified virus	0	0	0
***“dsDNA Virus*** c ***”***			
Marine Planktonic (virome)	36.1	94.5	NA
***Caudoviridae***	50.3	0.8	95.3
*Phycodnaviridae*	2.8	0.1	3.9
Marine Sediment (virome)	1.5	3.8	NA
Hot Spring (virome)	1.9	0.5	NA
*Mimiviridae*	1.4	0	0.3
*Iridoviridae*	0.4	0	0.4
Human Feces (virome)	0.1	0.2	NA
*Poxviridae*	0.1	0	0
***“Caudoviruses*** d ***”***			
*Myoviridae*	93.1	56.9	54.3
*Podoviridae*	5.1	30.6	27.6
*Siphoviridae*	1.5	11.1	17
Unclassified	0.05	0.3	1.2
Mixed	0.3	1.1	NA
***“NCLDVs*** e ***”***			
*Phycodnaviridae*	58.6	82.8	83.9
*Iridoviridae*	8	11.6	8.5
*Mimiviridae*	29.9	4.9	7.3
*Poxviridae*	2.3	0.5	0.3
*Ascoviridae*	0.3	0.1	0
*Asfarviridae*	1	0.1	0
***“Phycoviruses*** f ***”***			
Chlorovirus	46.1	80.4	80
Ostreococcus (OsV5)	43.3	17.5	18.7
Phaeovirus	1.9	0.4	0.8
Coccolithovirus	7	0.9	0.3
Prymnesiovirus (*P. globosa* virus)	NA	NA	0.05
Raphidovirus (*H. akashiwo* virus 01)	NA	NA	0.04
Mixed	1.3	0.8	NA

a % total protein sequences classified at highest (kingdom) taxonomic level per category (LF or VF).

b % total protein sequences characterized as viral per category.

c % of total of protein sequences characterized as dsDNA viruses comprising >0.1% in one category.

d % of total protein sequences characterized as Caudoviruses per category.

e % of total protein sequences characterized as NCLDVs per category.

f % of total protein sequences characterized as Phycoviruses per category.

n/a = not available.

According to phylogenetic characterization, the largest proportion of sequences from VF in the dsDNA virus category were most similar to sequences derived from environmental samples, including marine planktonic (∼95%) and marine sediment viral sequences (∼4%) (**Table 4**). Previous taxonomic characterization of marine viral metagenomes [6]–[8] suggests that the majority of viruses within these datasets are most similar to caudoviruses (i.e. tailed phages) and contain a large proportion of cyanophage-like sequences. Similarly, BLASTP-based comparisons of VF sequences against nr (NCBI) placed almost the entire dataset (∼95%) within the known *Caudoviridae* family, demonstrating the utility of including viral metagenomes in search databases (**Table 4**). Alternatively, phylogenetic characterization of LF viral sequences indicated that these were more similar to known caudoviruses (∼50%) rather than marine planktonic viruses (∼36%) suggesting inherent differences in population structure between the VF and LF (**Table 4**). These differences were more apparent at the family level where myovirus sequences were significantly more abundant in LF than VF, while podo- and siphovirus sequences were more abundant in the VF than LF. Overall, cyanophage-like sequences comprised a significant proportion of the Indian Ocean VF and LF caudovirus data based on BLAST and phylogenetic analyses; potentially reflective of the surplus of these sequences in reference databases. Sequences were most similar to phages infecting *Prochlorococcus* (P-SSM2, P-SSP7 and P-SSM4) and *Synechococcus* (S-PM2 and the Syn group of phages) (**Table S4**).

The VF and LF also differed with respect to the relative distribution of nucleocytoplasmic large DNA viruses (NCLDV), a group that includes the phycodna- and Mimi-viridae families that appear to infect eukaryotic organisms (**Table 4**). Although the sequenced strain of Mimivirus is known to infect amoebae [29], it has been proposed that marine Mimivirus-like sequences originate from viruses that infect a variety of marine protists including eukaryotic phytoplankton, specifically hapto- and prasinophytes [30]–[32]. Homologs to all 6 of the NCLDV families were detected in the LF and VF with different levels of relative abundance (**Table 4**). Overall, the LF had a larger proportion of sequences similar to the phyco- and Mimi-like viruses than VF. When restricting taxonomic evaluation to only NCLDV-related sequences, phycovirus homologs were more prevalent in the VF than the LF. The majority of VF phycovirus homologs were further classified as Chlorovirus while LF homologs were equally distributed between Chlorovirus and Ostreococcus virus OsV5 (**Table 4**). Mimivirus-like sequences were also more abundant in the LF than VF. The varying taxonomic distributions of viruses that likely infect bacteria (tailed phage) and eukaryotic phytoplankton (phyco- and Mimi-like viruses) between the VF and LF strongly suggests that size fractionation of marine microbial communities enriches for specific groups of viruses and significantly influences the distribution of viral families. This emphasizes the utility of sequencing across multiple size classes rather than just those designated as viral or cellular. The differential partitioning of viruses could be attributed to the morphology of the virus particle (for extracellular viruses), virus adsorption to host cells, active viral replication within infected host cells, and latent viral infection.

### Functional potential of Indian Ocean viruses

#### Comparison of VF and LF sequences

**Figure 2:**
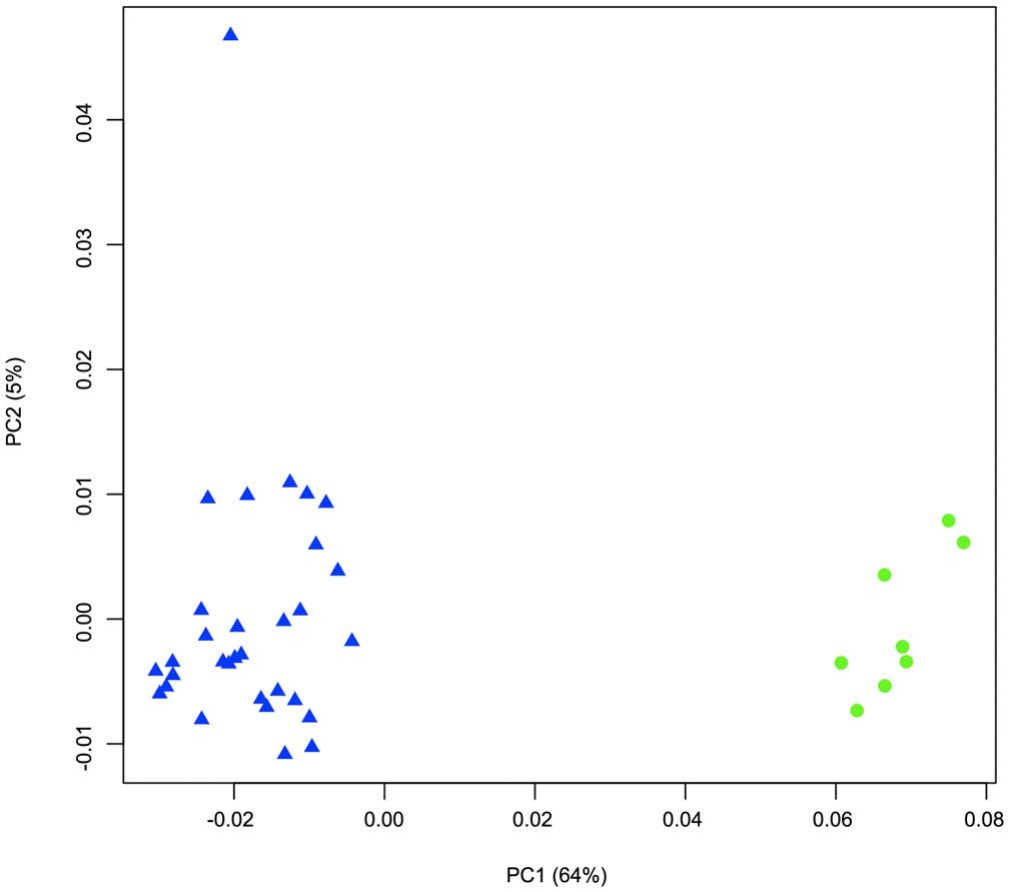
Principal component analysis (PCA) of the relative abundance of VF and LF viral sequences within protein clusters. Viral libraries are represented by the green circles and larger fraction libraries are represented by the blue triangles.

**Figure 3:**
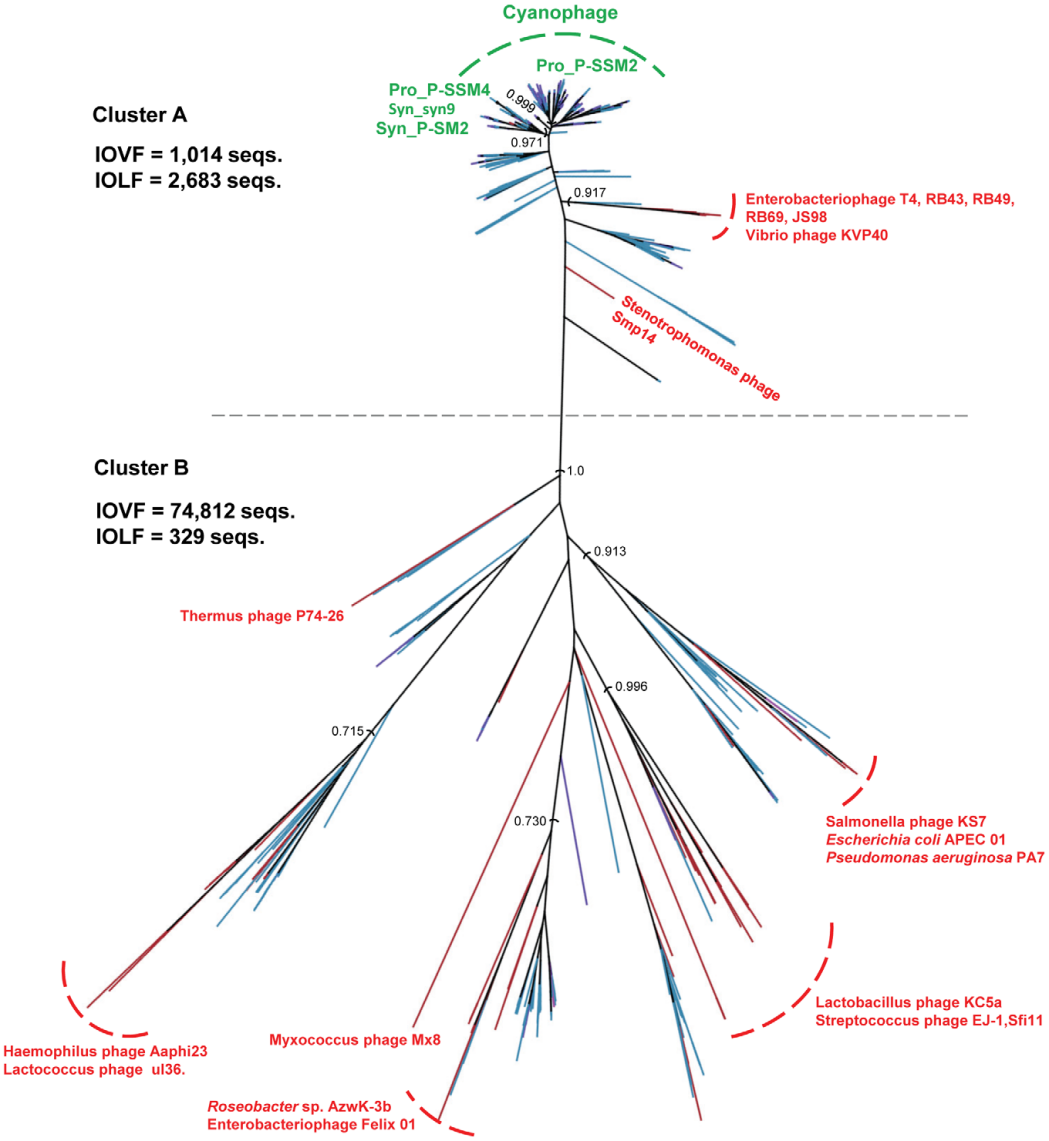
A phylogenetic tree built from amino acid sequences using FastTree for the gene encoding the large subunit terminase. Selected reference sequences are colored red and green. Representative Indian Ocean VF and LF sequences are colored pink and purple respectively. GOS Phase I sequences are colored blue. Confidence values are displayed on the tree.

To examine the gene function of viruses captured in this study, ORFs derived from sequencing reads were assigned to existing protein clusters that included the published GOS data as well as reference data [33]. The GOS-IO protein dataset consisted of all ORFs from the viromes (VF and LF). Statistical analyses were used to determine if significant relationships existed between the functional repertoire of viruses within the VF and LF (Principal Component Analysis, PCA), as well as the measured oceanographic environmental parameters (Canonical Correlation Analysis, CCA). Proportional abundances for all protein clusters were calculated by library and used in each analysis. PCA indicated that viruses within these two groups were functionally divergent (**Figure 2**). However, no significant relationship existed between viral function and environmental factors as measured by CCA (data not shown). A significant amount of the variance was accounted for by the first two components (69%), demonstrating that size fractionation partitions viruses with different functional potentials, likely related to the similar observation found by taxonomic evaluation. A PCA biplot was also used to identify protein clusters containing viral sequences that were driving the separation of the VF and LF groups. Of particular note, two different clusters containing the same viral protein, large subunit terminase, were over-represented; cluster A predominantly contained LF sequences (LF = 2,683; VF = 1,014), and cluster B mostly consisted of VF sequences (VF = 74,812; LF = 329). Terminases are viral enzymes consisting of a small and large subunit that enable the packaging of DNA into viral proheads [34]. The small subunit is responsible for DNA recognition while the large subunit performs several functions including DNA cutting, portal vertex docking and ATPase-mediated translocation of DNA [34]. A phylogenetic tree was created from representative sequences contained in these two clusters including Indian Ocean VF and LF, GOS Phase I and publicly available reference sequences. The tree was characterized by two phylogenetically distinct groups that effectively partitioned the two terminase clusters (**Figure 3**). The LF group, corresponding to cluster A, contained well supported clades with *Prochlorococcus* and *Synechococcus* cyanophage reference sequences in addition to environmental viral sequences, which could represent uncultivated cyanophage. The VF group, corresponding to cluster B, was more phylogenetically diverse, characterized by multiple well-defined clades containing mixtures of reference phage and environmental viral sequences. The reference phage in the cluster B group belonged to all three of the tailed phage families rather than just described T4-like myoviruses present in the LF group, similar to the taxonomic distribution of Indian Ocean VF sequences (**Table 4**). Terminase-mediated packaging of DNA is a conserved mechanism among diverse linear, dsDNA containing viruses [34]. Several studies indicate that the terminase large subunits share a common ancestry with other translocating ATPases including helicases and type I and III restriction endonucleases [35], [36] and that terminase phylogeny may be predictive of the nature of the ends of virus DNA (e.g. cohesive ends) [37]. Clading of the environmental virus sequences with T4-like reference phages in the cluster A group suggests that these viruses may possess terminally redundant, circularly permuted genomes that are packaged using a T4-like headful packaging mechanism. However, it is difficult to speculate as to the packaging mechanisms of viruses in cluster B since the nature of the DNA ends for the reference phage is unclear.

#### Pathway analyses

**Figure 4:**
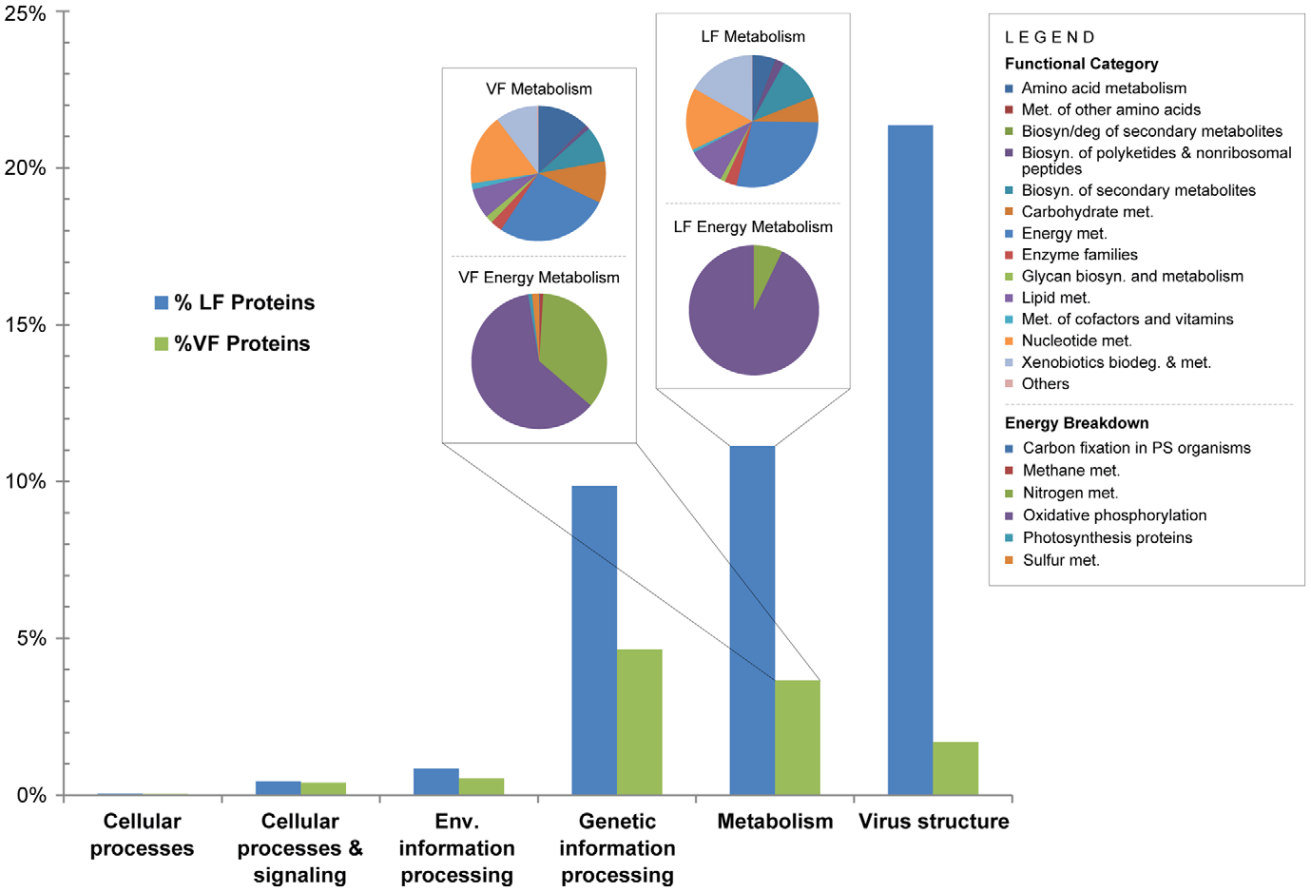
Functional characterization of Indian Ocean viral sequences from the viral and larger fractions of metagenomic data in the context of KEGG pathways. The inset pie charts represent the breakdown of the Metabolism super-pathway (top) and Energy metabolism pathway (bottom). The percentages of viral sequences attributed to the Energy metabolism pathway are indicated on the Metabolism pie charts.

Protein clusters containing viral sequences originating from the VF and LF were also categorized in the context of KEGG pathways, with the addition of a virus structure category, in order to assess the functional potential of Indian Ocean virioplankton. The majority of protein sequences from VF and LF were not mapped to a pathway and remained uncharacterized (VF = ∼80%; LF = ∼37%). This level of functional novelty was not unexpected due to high abundance of hypothetical proteins in each category (VF = ∼68%; LF = ∼50%). Smaller proportions of data were considered poorly characterized (VF = ∼3%; LF = ∼5%) or were not specific to a particular pathway (VF = ∼3%; LF = ∼8%). The remaining sequences were mapped primarily to the Virus Structure, Metabolism and Genetic Information Processing categories (**Figure 4**, **Table S5**). A heatmap of functional categories (**Figure S1**) further demonstrated the partitioning of VF and LF sequences as observed through PCA with differential clustering of the VF and LF. The vast majority of VF (∼79%) and LF (∼80%) sequences within the genetic information processing pathway were categorized as putative DNA replication, recombination and repair proteins (**Table S5**).

The largest proportion of viral sequences within the Metabolism pathway was attributed to energy metabolism, with a slight enrichment in the VF (**Figure 4**, **Table S5**). A relatively small proportion of these sequences from the VF and LF were classified into lower categories including nitrogen metabolism and oxidative phosphorylation. Although nitrogen metabolism genes have been previously documented in viral metagenomes created from a variety of environmental settings [11], the exact nature of the genes contributing to this pathway was unclear. Several studies have demonstrated that viruses infecting eukaryotic phytoplankton and zooplankton (likely to be retained in the LF) carry genes of either host or bacterial (i.e prey) origin [38]–[45]. However, the majority of these genes are involved with lipid, carbohydrate and protein metabolism and polyamine biosynthesis rather than nitrogen metabolism or oxidative phosphorylation [46]–[48]. Only VF sequences possessed enzyme commission (EC) numbers that could be linked directly to the KEGG nitrogen reduction and fixation pathway and these were examined in more detail. The majority of VF sequences within the nitrogen metabolism category were annotated as glutamate synthase (n = 98), which together with glutamine synthetase, comprise the GS-GOGAT pathway. This pathway facilitates the process of ammonium assimilation in phytoplankton [49] and is dependent on the availability of nitrogen compounds in the environment. The Indian Ocean is considered an oligotrophic water mass with very low concentrations of available nitrogen [50], and nitrogen concentrations measured in our samples were indeed indicative of a nitrogen-limited environment (**Table S1**). The presence of glutamate synthase genes suggest that viruses may play a role in nitrogen modulation and assimilation during the infection of host cells. Proteins involved in oxidative phosphorylation (OP) pathway were much more abundant than photosynthesis-related proteins, comprising ∼30% of VF and ∼11% of LF sequences within the energy metabolism category (**Figure 3**, **Table S5**); with 466 VF and 25 LF sequences possessing EC numbers. NADH dehydrogenase I subunit and inorganic diphosphatase were represented in both the VF (n = 255 and 53 respectively) and LF (n = 7 and 18 respectively) while the cbb3-type cytochrome C oxidase subunit I was only detected in the VF (n = 158). To the best of our knowledge, this is the first report of viral cytochrome C oxidase and inorganic diphosphatase genes in the marine environment. NADH dehydrogenase and cytochrome C oxidase are both components of the electron transport chain in bacteria, which is ultimately used to produce ATP. Viral type I NADH dehydrogenase genes were first reported by Alperovitch-Lavy and colleagues [51] and were detected through a combined analysis of GOS microbial scaffolds and long PCR amplification of viral fractions collected from the Pacific Line Islands [52]. Interestingly, the viral NADH dehydrogenase genes were co-localized on viral scaffolds (and amplicons) containing photosystem I and II genes suggesting that cyanophage encode this complex. A subsequent search of GOS scaffolds by Sharon and coworkers (2011) for viral auxiliary metabolic genes also revealed the presence of viral Type I NADH dehydrogenase subunits putatively involved in cyclic electron flow around PSI and respiration during viral infection. Again, these genes were attributed to cyanophage since the majority of scaffolds containing viral auxiliary genes that were examined in this study appeared to be related to know cyanophages [15]. It's possible that the viral NADH dehydrogenase genes observed in this study are of cyanophage origin due to the abundance of cyanophage- like sequences in the Indian Ocean data. However, the abundance of virus-SAR86 host predictions (discussed later) coupled with the presence of viral cbb3-type cytochrome C oxidases, which are only found in proteobacteria, suggests that viruses that infect heterotrophic bacteria may also be the source of these genes. The enzyme inorganic diphosphatase catalyzes the conversion of diphosphate (Ppi) to phosphate (Pi), which is needed for the production of ATP. Out of the three OP enzymes, inorganic diphosphatase was more evenly distributed between the VF and LF suggesting that a diverse group of viruses may carry this gene. If the viral version of inorganic diphosphatase is expressed and functional during infection, viruses could potentially contribute to host ATP production. This process could temporarily prolong the lifespan of the host and increase replication efficiency, analogous to viral NADH dehydrogenase and PS genes. An alternative hypothesis is that viral inorganic diphosphatase is used to produce Pi for incorporation into viral nucleic acids. Phosphate concentration in the marine environment is thought to influence virus production due to their inherently high nucleic acid to protein ratio [52]. The ability to influence the availability of phosphate during infection could maximize nucleic acid biosynthesis. Furthermore, a variety of phosphorous metabolism genes have been detected in the genomes of cultivated viruses that infect heterotrophic bacteria, cyanophage genomes [53], [54], as well as numerous viral metagenomes [11], [12], [55], suggesting that viruses have developed multiple strategies to address phosphate-limiting conditions. It is now well known that cyanophages carry photosynthesis (PS) related genes, including those associated with photosystems I and II [14], [56]–[59], and the presence of viral PS genes has been documented in numerous marine metagenomic studies [7], [12], [13], [15], [18]. However, only a small proportion of VF sequences (0.42%) could be mapped to proteins involved in photosynthesis based on KEGG classification of protein clusters. A direct BLAST analysis of VF and LF sequences using PSI and PSII genes collected from cyanophage genomes (PSII) as well as *Prochlorococcus* and *Synechococcus* (PSI) (**Table S6**) did reveal the presence of additional viral PS genes. The PSII genes psbA and psbD (total = 6,877) far outnumbered the PSI genes that were previously noted in the marine environment including psaA, psaB, psaC, psaD psaE and psaK (total = 371) (**Table S7**). Viral PSI genes were also noted in previous analyses of GOS microbial metagenomic data including 17 samples collected from the Indian Ocean [14], [15]. It is hypothesized that viral PSI components may facilitate electron donation from alternative sources other than plastocyanin to the PSI of their hosts, thereby increasing ATP generation for replication [14]. The discrepancy in the abundance of viral PSII versus PSI genes in the Indian Ocean data suggests that cyanophage may benefit more from carrying PSII genes, which have been shown to supplement photosynthesis in culture [60], [61].

Carbonic anhydrase (CA) enzymes were also present in the viral data (VF = 24; LF = 1). CA is responsible for catalyzing the conversion of carbonic acid to CO2 and is utilized by a diverse group of marine phytoplankton as part of their inorganic carbon concentration mechanism (CCM) to support photosynthetic carbon fixation [62]–[64]. Again, this is the first report of viral CAs in the marine environment to the best of our knowledge. Together with the PSI and PSII genes, the presence of these viral CAs suggests that the repertoire of viral-encoded photosynthesis-related genes is broader than previously recognized.

### Identification of potential virus-host relationships

There is a growing body of evidence based on analyses of fully sequenced viral genomes that phage tend to mimic the polynucleotide sequence composition of their hosts [65]–[68]. On this evidence, we developed the first classification method to predict the taxonomy of bacterial hosts for uncharacterized viral metagenomic sequences that does not rely on homology or sequence alignment; rather it compares sequence composition between viruses and bacteria. Briefly, our MGTAXA method involves three steps [69]: (I) trains one Glimmer Interpolated Context Model (ICM) [70] for every taxonomic node represented by at least one available bacterial reference sequence; (II) scores each metagenomic viral sequence against all models; and (III) picks the model with the highest score as representing the taxonomy of the putative host. This follows the approach of the Phymm bacterial classifier [21] where it was used to assign taxonomy to bacterial metagenomic sequences. The benchmarking of this classification scheme on temperate phages resulted in 97% accuracy at the phylum level and 89% at the genus level of the predicted host taxonomy with 2% rejection rate of the testing samples, and on all phages regardless of replication strategy - in 76% accuracy at the phylum level and 50% at the genus level with 6% rejection rate (see Methods S1, for detailed method description and benchmarking protocol). If the method were selecting candidate hosts from all bacterial genomes in RefSeq in an unbiased way, a randomly generated genus-level assignment would have an average accuracy of 0.2%.

Two approaches were taken to predict the bacterial hosts: 1) direct assignment – the ICMs trained on RefSeq (NCBI) genomes and 2) a transitive assignment – the hosts were predicted using ICMs trained on large metagenomic bacterial scaffolds (>100 kb) from the cellular fraction, to which a bacterial taxonomy was in turn assigned based on scoring against RefSeq ICMs (see Methods S1, for details on cellular fraction contig selection and taxonomic assignment). The essence of the transitive assignment process is that it restricts the model set to the bacterial genomes that are most abundant in a given metagenome (at least those that assembled into large scaffolds) before using that model set for host assignment. The value of the transitive method is that it recruits viral contigs to microbial scaffolds obtained from the same environment. On the other hand, depending on the dynamics of the phage-host system as well as sequence variability, the hosts for the assembled viruses might not necessarily be the best assembled microbes. In that case, the direct assignments can still be more accurate and informative. Thus, we consider these two approaches to function synergistically in the designation of virus∶host classifications.

**Figure 5:**
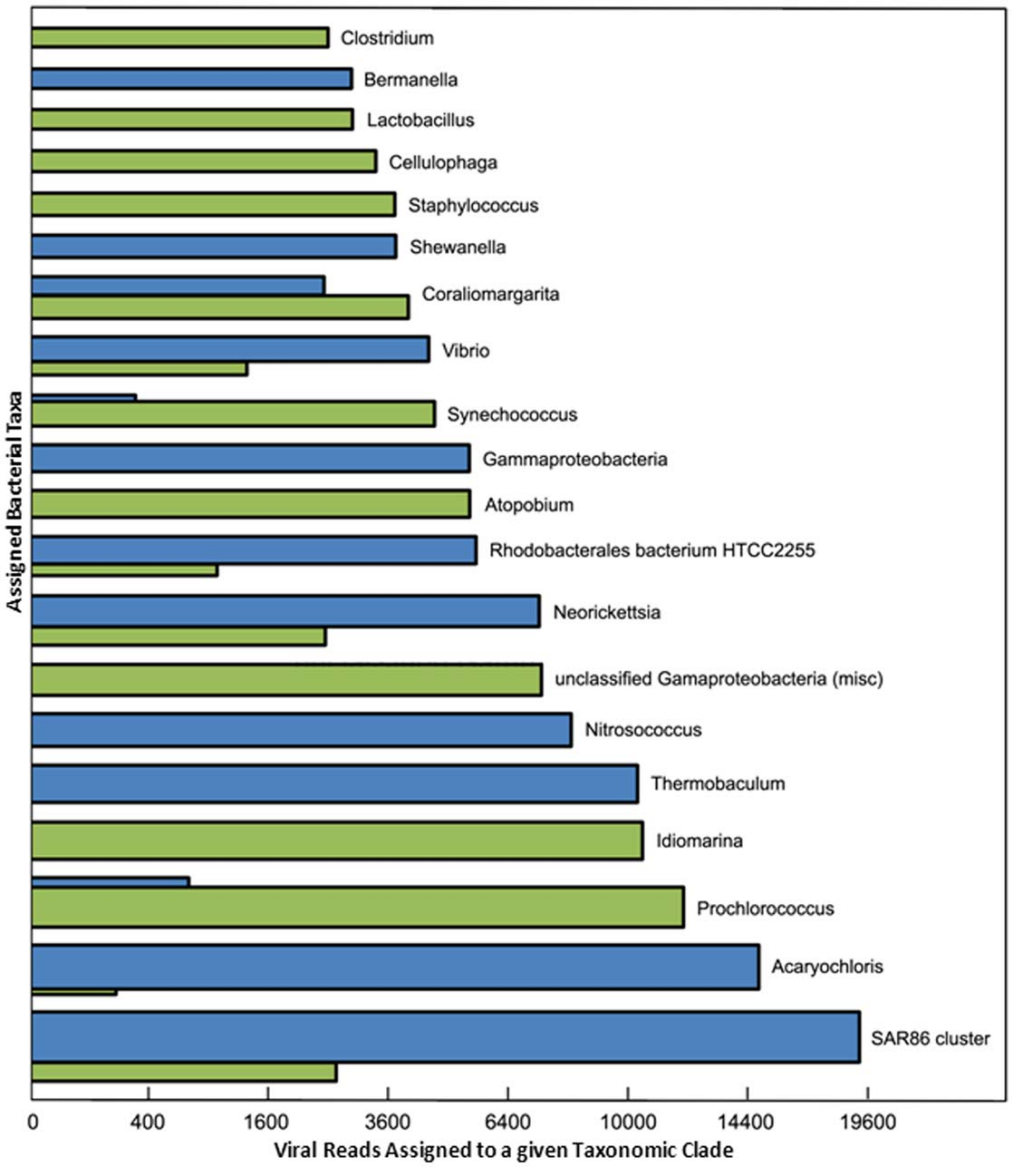
Bar chart demonstrating the predicted taxonomy of bacterial host genera for assembled Indian Ocean viral metagenomic data. The area of each bar is proportional to the total number of viral reads contained in the contigs assigned to a particular host genus. Direct assignments are colored green and transitive assignments are colored blue. Direct assignments were made by assigning viral contigs to the host models built on NCBI RefSeq genomes. Transitive assignments were made by assigning viral contigs to models built on large bacterial contigs, and then assigning taxonomy to these bacterial contigs (as described in the text). Twenty top taxa in pairs of green and blue are shown according to the maximum count from either direct or transitive assignment method, respectively. If no bar is shown for one of the methods, the count was zero.

The method was subsequently applied to all 5 Kb or longer contigs assembled from the Indian Ocean 454 viral libraries. **Figure 5** demonstrates the results of MGTAXA predictions of putative host taxonomy for the viruses present in the Indian Ocean metagenomic dataset. We found that even when all RefSeq bacterial genomes were used for host prediction (i.e. direct assignment) there was a preferential selection of microbial taxa that are indigenous to the marine environment including *Prochlorococcus* and *Idiomarina*. Assignments to NCBI unclassified Gammaproteobacteria (which encompasses diverse marine isolates including the uncultivated SAR86 cluster), and to a lesser degree, the SAR86 cluster itself were also prevalent [71]. This behavior further supports the validity of our host prediction method already demonstrated on the compiled benchmark. The transitive assignments redistributed the number of assigned viral reads per contig towards fewer overall bacterial genera, generally focusing on those that demonstrated the highest abundance in microbial metagenomic data (**Table S8**) such as the SAR86 cluster (119 scaffolds) and *Rhodobacterales* HTCC2255 (33 scaffolds). This result was expected by the design of the transitive assignment methodology. However, this trend was not universal, as demonstrated by the number of *Prochlorococcus* assignments for which 38 bacterial scaffolds were assigned (**Figure 5** **; Table S8**). The viral contigs that were assigned to these putative host taxa by the direct method were largely reassigned to several different bacterial genera by the transitive method (**Table S9**). Transitive assignments to Candidatus Pelagibacter and the SAR11 cluster were also sparse despite the prevalence of bacterial scaffolds attributed to these organisms (87 and 33 respectively) (**Table S8**). The top predicted host for Indian Ocean viral assemblages using transitive methodology was the SAR86 cluster. The SAR86 cluster encompasses a group of uncultivated, proteorhodopsin-containing Gammaproteobacteria [72] and can comprise a significant portion of the microbial communities in various marine environments [73], [74]. Since the bacterial members of the SAR86 cluster remain wild, no phages infective for these organisms have been reported. However, it is unlikely that these microbes are completely resistant to viral infection. Indeed, our results indicate otherwise and suggest that SAR86 virus-host interactions prevailed in the tropical Indian Ocean at the time of sampling. Due to the lack of specific SAR86 virus isolates and their corresponding genomic information, none of our viral metagenomic data could be specifically assigned to these putative viruses through our taxonomic analyses. This limitation further highlights the benefit of the homology-independent predictions of host taxonomy by revealing previously unknown, yet potentially significant virus-host relationships. Cyanobacteria within the Acaryochloris genus became the second most abundant predicted host for viruses in the Indian Ocean through transitive assignment despite the fact that only 10 bacterial scaffolds were assigned to this organism. Acaryochloris exists as either a free-living organism or as a symbiont of higher organisms (including macroalgae and ascidians) and is unique among the cyanobacteria since its main photosynthetic pigment is Chl*d* rather than Chl*a* [75], [76]. Two Acaryochloris phage have also been isolated and their genomes sequenced and recently described by Chan and coworkers [77] who documented the unique presence of mitochondrial DNA polymerase. The authors of this study found homologs of this phage-encoded gene in the GOS microbial data, including the Indian Ocean, further suggesting that these cyanophage (and their hosts) are present in this environment. It's possible that these viruses were more abundant in the Indian Ocean at the time of sampling than indicated by our analyses with related sequences not receiving a definitive taxonomic assignment as discussed previously.

### Summary

This is the first study to examine the Indian Ocean virome using holistic metagenomic approaches. Since our analyses were not constrained to the “viral fraction” of samples, we were able to gain a much more comprehensive understanding of virus diversity, total gene complement and functional potential as well as virus-host relationships. Significant taxonomic differences were evident between viruses represented in the VF versus the LF. An enrichment of cyano-myoviruses and viruses that likely infect eukaryotic phytoplankton or heterotrophic protists was found in the LF; while podo- and siphoviruses were prevalent in the VF. Similarly, notable differences in functional potential were evident by the distribution of abundances within metabolic pathways. The presence of putative viral genes potentially involved in nitrogen metabolism, carbon fixation and oxidative phosphorylation suggests that viruses infecting autotrophic and heterotrophic microbes may influence host cell physiology through diverse mechanisms in the Indian Ocean. Predicted virus-host relationships suggest that members of the SAR86 cluster and the cyanobacteria Acaryochloris and *Procholorococcus* represent the dominant hosts for viruses in the Indian Ocean, providing insight into the types of viruses that putatively possess these metabolic capabilities.

## Materials and Methods

### Sample and metadata collection & size fractionation

Surface water samples (∼400 L) were collected from 17 sites from the tropical Indian Ocean between August and October, 2005 aboard the S/V Sorcerer II. Two additional sites, GS148 and GS149 (∼200 L), were sampled off the island of Zanzibar, Tanzania using alternate vessels. All field studies conducted within the EEZ of foreign nations received Marine Research Permits as required under the U.N. Convention on Law of the Sea, and as required, separate agreements to access genetic resources. The locations were not privately owned or protected in any way and the field studies did not involve endangered or protected species. A YSI (model 6600) was used to measure the physical environmental parameters including water temperature, salinity, dissolved oxygen and sample depth. Sub-samples were collected for dissolved nutrient analyses as described previously [23] and were processed by the Virginia Institute of Marine Sciences (VIMS) Analytical Service Center. The microbial community was first passed through a 20 µm Nytex pre-filter and then size fractionated by serial filtration through 3.0 µm, 0.8 µm and 0.1 µm membrane filters (Pall Life Sciences, East Hills, NY). Filters were preserved and stored as described previously [23].

### Virus concentration & purification

The viral fraction of water samples (i.e. <0.1 µm) was concentrated using tangential flow filtration (TFF) as described previously [22]. Viral concentrates (VCs) were cryo-preserved through the addition of glycerol (10% final concentration) and frozen at −20°C. VCs were transferred to and stored at −80°C upon return to the J. Craig Venter Institute (JCVI) until further processed. VCs were further concentrated, treated with nuclease to remove free DNA and pelleted through a sucrose cushion as previously described [22]. To check for cellular contamination a 16S rRNA gene PCR was used with positive (*E. coli* cells) and negative (DEPC water) samples. Gel electrophoresis was performed on 2 µl (of 50 ul total reaction volume) on a 0.8% agarose gel stained with SybrGold (Invitrogen). If no discernible 16S rRNA gene product (∼1500 bp) was visualized the samples were further processed through DNA extraction.

### DNA extraction, library construction, sequencing and post-processing of data

Methods describing DNA extraction from filters, construction of clone libraries, template preparation, and automated cycle sequencing can be found in Rusch *et al.*, 2007. Viral DNA was extracted from purified VCs and modified linker amplified shotgun libraries (LASLs) were constructed as described previously [22]. In addition to Sanger libraries, 454 Titanium libraries were prepared from amplified viral DNA (LASLs) and pyrosequenced at JCVI. Briefly, genomic DNA was fragmented and size-selected to a range of 500–800 bp. Linkers were ligated to DNA fragments for use as priming sites during subsequent amplification reactions. Three replicate amplification reactions were completed using 15 total cycles to reduce biases and amplification of potential cellular contamination from kit reagents. Adaptors were ligated onto the fragments and used as priming sites for emulsion PCR. Amplified samples were purified using AMPure beads to remove small DNA fragments and sequencing was performed using the 454 GS FLX Titanium sequencing platform. Viral metagenomic sequences were trimmed of any linker sequence left over during LASL production. Additionally, artificial replicates were screened for and removed from all 454 data using an approach described by Gomez-Alvarez (2009) [78].

### Identification of viral sequences in the filter fractions of data & taxonomic profiling

The Automated Phylogenetic Inference System (APIS) was used for the taxonomic classification of viral predicted proteins as well as the identification of viral proteins within the larger size classes of data [25]. APIS automated the process of calculating sequence similarity, alignment, and phylogenetic inference for each protein in a given dataset. Each predicted protein was compared to an in-house curated database (phyloDB), which consists of proteins from complete (or nearly complete) genomes and selected Sanger-sequenced viral metagenomes, using BLASTP. Full-length sequences of significant BLASTP hits were retrieved and then a multiple alignment was generated using MUSCLE. From this alignment, a neighbor-joining tree was produced using QuickTree [79] to determine the phylogenetic placement of the query sequence by comparing the taxonomic classification of the sequence(s) that clade with the query. If the taxonomic information differed among these clading sequences, this was noted and the classification of the query was limited to the higher taxonomic ranks where they were in agreement. To identify viral sequences from the larger size class (0.1–20 µm) of organisms, all proteins classified via APIS as viral at the Kingdom level were used in further analyses.

A BLAST-based approach was used, which is part of the Viral Metagenome Annotation Pipeline (VMGAP) [80]. Environmental sequences were compared against NCBI AllGroup.niaa database (BLASTP) using an e-value of < = 1e−10 and identity > = 50%, the top hit was noted and taxonomic information transferred to each metagenomic protein.

### Functional characterization of viral sequences

#### ORF prediction and viral annotation

Open reading frames were predicted as described previously [81], and were based on a combination of naïve 6-frame translations and MetaGeneAnnotator [82], an *ab initio* gene finder program. The predicted protein coding sequences were then annotated using the Viral Metagenome Annotation Pipeline (VMGAP), described by Lorenzi and coworkers [80].

#### Annotation of protein clusters and KEGG classification

ORFs predicted on 454 sequence reads were mapped to protein clusters as follows. RPS-BLAST was first used to compare each protein sequence against a database of Position Specific Scoring Matrices (PSSM) representing clusters containing more than 20 proteins. Then, proteins were assigned to clusters based on the PSSM that gave the highest bit score with an e-value ≤1×10−^3^. For those protein sequences that did not produce any significant hit against the PSSM database, a BLASTP search was conducted against a database of proteins belonging to clusters containing fewer than 20 members. Proteins were then assigned to clusters based on the hit having the lowest e-value with at least 40% identity and 70% coverage. Protein sequences that did not result in any significant hit remained unassigned.

Annotation was performed on all protein clusters [33] from the individual predicted protein annotations within each cluster. All protein annotations were counted within, and across, all clusters and an uncorrected p-value was calculated using Fisher's exact test indicating the probability of random association of each annotation with each cluster. The annotation, description, coverage and p-value were given for each of the 3 best annotations. For clusters that did not receive annotation by this method, a different strategy was taken based on additional searches and relative ranking of the results (Methods S1, for details). To bin protein clusters into KEGG classes, all cluster names having a match that was 100% identical to any KEGG class definition were assigned to the corresponding three levels of classification from the KEGG Pathway Database. When more than one possible classification class were available at a particular level, all classes representing <75% of that level were labeled “unspecific”. Clusters without any direct KEGG association were examined for the presence of specific keywords and then classified following the same three-level classification. Otherwise, clusters were binned as unclassified for each level.

#### Large subunit terminase phylogeny

Environmental sequences and references were retrieved from two proteins clusters, both containing putative genes encoding terminase enzymes. The average length of all sequences was calculated and sequences below 40% of the average were removed. Non-viral GOS Phase I sequences were reduced through clustering and only the representative sequence used in subsequent steps. Existing hidden Markov model (HMM) profiles, including fragment HMMs, obtained in cluster annotation or peptide annotation were identified and the HMM that accounted for the majority of viral sequences was selected for alignment using HMMER (http://hmmer.org/). The multiple alignments were processed through a gap-filtering step to remove sequences that contain 60% or more gaps. Phylogenetic trees were constructed using the program FastTree [83] and processed using Archaeopteryx (http://phylosoft.org/archaeopteryx/).

### Statistical analyses

Principal Component Analysis (PCA) and Canonical Component Analysis (CCA) were performed using the R statistical program. For PCA, library proportional abundances of viral sequences for each protein cluster were calculated and used to build the centered matrix. For CCA, oceanographic metadata was included to assess correlation between these and protein cluster proportional abundances.

### Metagenomic assembly

The Sanger and 454 data from each viral library were assembled independently using the Newbler GS De Novo Assembler, version 2.3, with a minimum identity threshold of 86%. In addition to virus-specific assemblies, a comprehensive global assembly was also conducted on all Sanger reads produced from Phase I of GOS and all Indian Ocean size fractions as described by Rusch et al. 2007 using the Celera Assembler version 5.3 with a minimum identity threshold of 86%. A minimum overlap length of 40 bp was used for all assemblies.

### Genotype diversity estimates

A local command line version of the PHACCS program [26] was used to estimate viral genotypic diversity. The 454 datasets were randomly sub-sampled 8–10 times prior to assembly to approximate the same amount of base pairs as the corresponding Sanger datasets and the resulting contig spectrums were used as input. The average genome size used was 50 Kbp for all, except where the 454-subsampling used additional genome sizes to assess the effect on diversity estimates, and the minimum overlap was 40 bp. Averages and standard deviations were calculated in Excel.

### Virus-host classification

As this is the first report of an alignment-independent method that aims to predict the virus∶host relationships in the metagenomic datasets, detailed methodology and benchmarking is reported in Methods S1. Briefly, sequence composition similarities were used to bin viral contigs with 1) bacterial hosts from RefSeq and 2) the cellular fraction metagenomic sequences corresponding to this dataset. Hosts were described at the genus taxonomic level; if the lowest level reached for assignment was order or higher, the viral contigs were labeled as ‘unassigned’. The implementation parallelized for a distributed computing cluster is available as part of our open-source software package MGTAXA [69].

### Accession numbers

All Sanger-generated viral data was submitted to the NCBI Trace Archives. These include the raw reads from viral metagenomes as well as the predicted protein sequences extracted from the larger size fractions of data. All 454-generated viral data was submitted to the NCBI Short Read Archive (SRA), corresponding to accession numbers SRX096024, SRX096023, SRX096025, and SRX096299. The microbial and viral assemblies used for the analysis of virus-host classification were submitted under NCBI's Project ID 19733.

## Supporting Information

Methods S1 **Additional methods including, i) Alternative cluster annotation, ii) Identification of viral photosynthesis genes, iii) Classification of Virus∶Host relationships.**
(DOCX)

Figure S1 **Heatmap showing delineation of viral sequences from large vs. viral fraction.** Abundance of sequences from each site within selected functional groups at the level-2 classification of Gene Ontology (GO) was used to generate heatmap. Hierarchical clustering of sites indicates a grouping of large (blue) versus viral (green) fraction. Functional groups are color-coded as follows: cellular processes (purple), cellular processes and signaling (blue), environmental information processing (green), genetic information processing (yellow), metabolism (orange), phage structure (red).
(TIF)

Table S1 **Indian Ocean sample characteristics and oceanographic metadata.**
(XLSX)

Table S2 **Viral sequences retrieved from the larger size classes.**
(XLSX)

Table S3 **Assembly statistics for Indian Ocean viral metagenomic libraries.**
(XLSX)

Table S4 **Top five most abundant cyanophage homologs based on taxonomic assignments.**
(XLSX)

Table S5 **Functional characterization of VF and LF viral sequences by KEGG category.**
(XLSX)

Table S6 **List of reference sequences and genomes used to identify Indian Ocean viral photosystem genes.**
(XLSX)

Table S7 **Viral photosystem genes present in the Indian Ocean metagenomic data.**
(XLSX)

Table S8 **GOS Bacterial Scaffold Taxonomic Assignments based on MGTAXA.**
(XLSX)

Table S9 **Re-distribution of viral contigs to bacterial taxa from direct to transitive assignments.**
(XLSX)

Table S10 **NCBI RefSeq prokaryotic genomes used for building reference models.**
(XLS)

Table S11 **Benchmarking accuracy for predicting the host taxonomy.**
(XLSX)

Table S12 **Benchmarking set of virus∶host pairs compiled from NCBI RefSeq.**
(XLS)

Table S13 **Summary counts for the benchmark virus∶host pairs in Table S12.**
(XLSX)
