# Supplementary material for: Metagenomic Exploration of Viruses throughout the Indian Ocean
Source: PLoS One. 2012 Oct 17;7(10):e42047. doi: 10.1371/journal.pone.0042047 (PMC3474794; doi:10.1371/journal.pone.0042047)
Supplement: Methods S1 — Additional methods including, i) Alternative cluster annotation, ii) Identification of viral photosynthesis genes, iii) Classification of Virus∶Host relationships. (DOCX) [file pone.0042047.s001.docx]

**Methods S1:**

1. **Alternative cluster annotation:**

For clusters that did not receive functional annotation based on the method described in main text, an alternative three-step approach was taken to establish cluster annotation. During the first step, product names from all proteins belonging to public repositories were collected, cleaned from all uninformative words such as “similar”, “protein”, “putative”, “like”, etc…, and then grouped if they shared at least 80% identity. In those cases where proteins were part of an entry in the KEGG Orthology Database, product names were replaced by their respective KEGG definition line before cleaning. Afterwards, name groups were ranked based on the number of names they contained in decreasing order. Finally, the most frequent name from the group having the highest rank was selected as the cluster name. In those cases where the chosen name was “hypothetical protein”, then the most frequent name from the second best ranked group was picked as the cluster name, as long as the names from that group represent at least 75% of the total number of names excluding the best ranked group. In Step 2, PFAM/TIGRFAM HMM, ACLAME HMM and CDD PSSM searches were performed on all predicted proteins from the same cluster and assigned a product name to each protein based on the following priorities in decreased order: global PFAM/TIGRFAM hit (e-value ≤ 1x10^-5^, HMM %coverage ≥ 50%); ACLAME hit (e-value ≤ 1x10^-3^, %coverage ≥50%); CDD hit (e-value ≤ 1x10^-7^ %coverage ≥ 70%); local PFAM/TIGRFAM hit (e-value ≤ 1x10^-5^, HMM %coverage ≥ 50%); cluster name from Step 1. Finally, during Step 3, predicted protein names were grouped and ranked as indicated above, and the final cluster name was assigned from the best or second best ranked group of names as described for Step 1. Clusters without any evidence at all were named as “unknown protein”.

1. **Identification of viral photosynthesis genes:**

Putative cyanophage photosystem homologs were identified by comparing the Indian Ocean viral sequences against photosystem I and II genes from a series of reference cyanobacterial genomes and phages (**Table S6**) known to encode the relevant genes. BLASTP was used with a e-value cutoff of 1x10^-4^.

1. **Classification of Virus:Host relationships**:

*General methodology:*

The classification method used, MGTAXA [1], has three initial steps similar to the Phymm bacterial classifier [2]: (I) trains one Glimmer Interpolated Context Model (ICM) [3] for every taxonomic node represented by at least one available bacterial reference sequence; (II) scores each metagenomic viral sequence against all models; and (III) picks the model with the highest score as representing the taxonomy of the putative host (as NCBI taxonomy ID). Note that during scoring, the ICM estimates the conditional probability of observing the next nucleotide given a context of *k* preceding nucleotides already observed in the input sequence (*k=11*). One consequence of this is that a phage sequence can still score “high” for a given model even if a simpler compositional metric such as G+C content is different between the phage and its host that generated the model. Unlike in Phymm, ICMs were also trained for all internal nodes of the NCBI taxonomic tree (e.g. family, order, phylum etc). The training sequences for the internal nodes were collected by a balanced recursive sampling procedure to reduce a disproportionate influence from any sub-tree with multiple sequenced strains. We use the internal node ICMs to increase the specificity of host assignments. We mark viral contigs as “unassigned” if the predicted host taxonomic rank is above the order level.

In addition to the assignment of the host taxonomy for viruses, essentially the same method is used for assigning taxonomy to metagenomic bacterial sequences as needed for the “transitive” host prediction described below.

The open-source implementation can be executed either in parallel on a distributed computational cluster or sequentially on a single workstation.

*Direct host assignment of viral contigs using RefSeq:*

The virus-host classifier trained Interpolated Context Models (ICMs) for all bacterial genomes residing in NCBI RefSeq (downloaded in November 2010) as well as mixtures of these genomes for the higher order clades and two SAR86 genomes partially assembled from the GOS microbial metagenomic data [4]. The total number of reference organisms was 2,020 including the assembled SAR86 scaffolds, comprising 463 genera. Thus, if we were to assign host genus taxonomy randomly, we would get 0.2% per-sample accuracy. The SAR86 model was used to make assignments to the species-level node "uncultured SAR86 cluster bacterium" in the NCBI taxonomic tree (NCBI taxonomy IDs, accession numbers and names of all references are provided in **Table S10**).

*Transitive host assignment using cellular fraction (Global Ocean Sampling – Indian Ocean; GOS-IO) metagenomic assemblies:*

As described in the main text, for transitive assignments we first assigned viral contigs to hosts represented by large bacterial metagenomic scaffolds from a cellular fraction, and then assigned bacterial taxonomy to those scaffolds. The selection of reference scaffolds for the GOS-IO ICMs was carried out as follows: (I) APIS read-level assignments were projected onto corresponding assembled scaffolds and the majority call was made at every major taxonomic rank to create an APIS assignment for the entire scaffold; (II) only scaffolds longer than 100 Kb were selected in order to have enough sequence to train each ICM; and (III) only scaffolds that did not have any of the clade levels assigned to the viral tree were selected. This consensus gene homology classification was used to select bacterial and archaeal scaffolds in favor of a composition-based method due to similarities in phage/host sequence composition that make it hard to distinguish viruses from their bacterial or archaeal hosts. Taxonomy was assigned to the resulting scaffolds using our MGTAXA classifier with RefSeq ICMs. According to the benchmarking protocol described below, the genus-level assignment accuracy for novel species is estimated to be 85% (91% for family and 95% for order taxonomic levels, with the full set of benchmarking results provided in **Table S11**). After training GOS-IO ICMs on these scaffolds and assigning them as putative hosts to the viral contigs, the predicted host taxonomy of each viral contig was produced.

*Benchmarking taxonomic classification of the bacterial scaffolds for the “transitive” host prediction protocol:*

We have generated testing samples by extracting fragments of 100Kbp from all genomes (excluding plasmids; phages if found in the original genome sequencing project were already excluded by the design of upstream RefSeq microbial FASTA files). All sequences for a given genome were concatenated to build a model. To evaluate the accuracy at the increasing degrees of taxonomic novelty, we adopted the protocol used in Phymm [2]. Specifically, for each genome used for testing, we excluded from the classification process all models from progressively higher taxonomic ranks containing this genome, as well as all synthetic models in the lineage of this genome. For each exclusion level, we evaluated the prediction accuracy at the main ranks (genus, family, order, class, phylum), generating a matrix of accuracy levels. Additionally, we have generated the accuracy at different levels when no model exclusion was made, simulating a situation when the target genome is present in the reference database. The availability of at least two sister clade genomes was required to consider a given genome for testing classification accuracy under a given taxonomic rank when the model exclusion protocol was used, **Table S11** reports mean per-sample accuracy (defined as number of true positives divided by the number of samples).

*Benchmarking the prediction of host taxonomy for viral sequences:*

The benchmark was set up by collecting all prokaryotic host records from Genbank viral files from the same version of RefSeq as reported for the bacterial classification benchmarking described above. In the absence of an explicit “host” record, we parsed the names of the viruses and matched them to the names of the cellular organisms. We only kept the records which had host taxonomy defined at a level of species (**Table S12**). We report NCBI taxonomy IDs and accession numbers for the viruses paired with the corresponding host taxonomy IDs and names, with two extra fields that we discuss further in the text. **Table S13** provides aggregate counts for the benchmark dataset.

For benchmarking, the viral sequences were shredded into 5Kbp fragments, and the fragments were scored against the microbial models built for all prokaryotic genomes in RefSeq as described above for the bacterial classification. To evaluate classification performance, we labeled each testing sample with the taxonomy of its host from our benchmark set, and then applied the evaluation protocol described above for the bacterial classification, with the host prediction results provided in (**Table S11-B)** and clade exclusion performed to the species level. The metrics are calculated after removing the samples rejected due to a criterion that the host assignment must be at the order level or below (corresponding percentages provided in column “reject”). Note that unlike in the bacterial classification case, in the settings when no model exclusion is performed, most of the virus:host database pairs might still not have the exact true host genome represented in the model set. This is because the available host information is rarely that specific.

To test if the accuracy is higher if the phage is known to propagate through the lysogenic cycle, we took the following steps to select a reliable temperate phage subset from our benchmark. First, we selected all phage:host pairs that had designated a specific host genome (in other words, strain), as opposed to merely more generic information about a species. We then reviewed the original publications that established the association between the phage and the host strain for information about the phage cycle. The majority of phages with such strain-specific host records turned out to be temperate. As documented in **Table S12**, the field ‘Specific Host Genome Known’ is set to TRUE if the host strain is known, and the field ‘Phage Cycle’ provides the information we collected from the literature (referenced by PUBMED Central ID where available or by PUBMED ID otherwise) . **Table S11-A** shows the benchmarking accuracy for this temperate subset. We observe a dramatic improvement compared to the entire benchmarking set when considering temperate phage:host pairs. A striking feature is that the genus-level prediction accuracy is still 85% even when the entire true host species is excluded from the model set, not even the specific host strain. In other words, for temperate phages, the prediction accuracy is still high even when the available reference genomes belong to sister species of a true known host. Note that that we can observe a phylum level accuracy slightly smaller than the preceding class-level accuracy. This is because not every main rank is defined for every bacterial genome in the NCBI taxonomic tree, and so the results from some samples contribute to only some of the ranks in the aggregate accuracy table.

**Supporting Information References:**

1. MGTAXA. Available at: <http://andreyto.github.com/mgtaxa/>. Accessed 2011 May.

2. Brady A and Salzberg SL (2009) Phymm and PhymmBL: metagenomic phylogenetic classification with interpolated Markov models. Nat Methods 6: 9 673-676.

3. Delcher AL, Harmon D, Kasif S, White O and Salzberg SL (1999) Improved microbial gene identification with GLIMMER. Nucleic Acids Res 27: 23 4636-4641.

4. Dupont CL, Rusch DB, Yooseph S, Lombardo MJ, Alexander Richter R*, et al.* (2011) Genomic insights to SAR86, an abundant and uncultivated marine bacterial lineage. The ISME J 6: 1186-1199.
