# Supplementary figures and images for: Metagenomic Exploration of Viruses throughout the Indian Ocean
Source: PLoS One. 2012 Oct 17;7(10):e42047. doi: 10.1371/journal.pone.0042047 (PMC3474794; doi:10.1371/journal.pone.0042047)

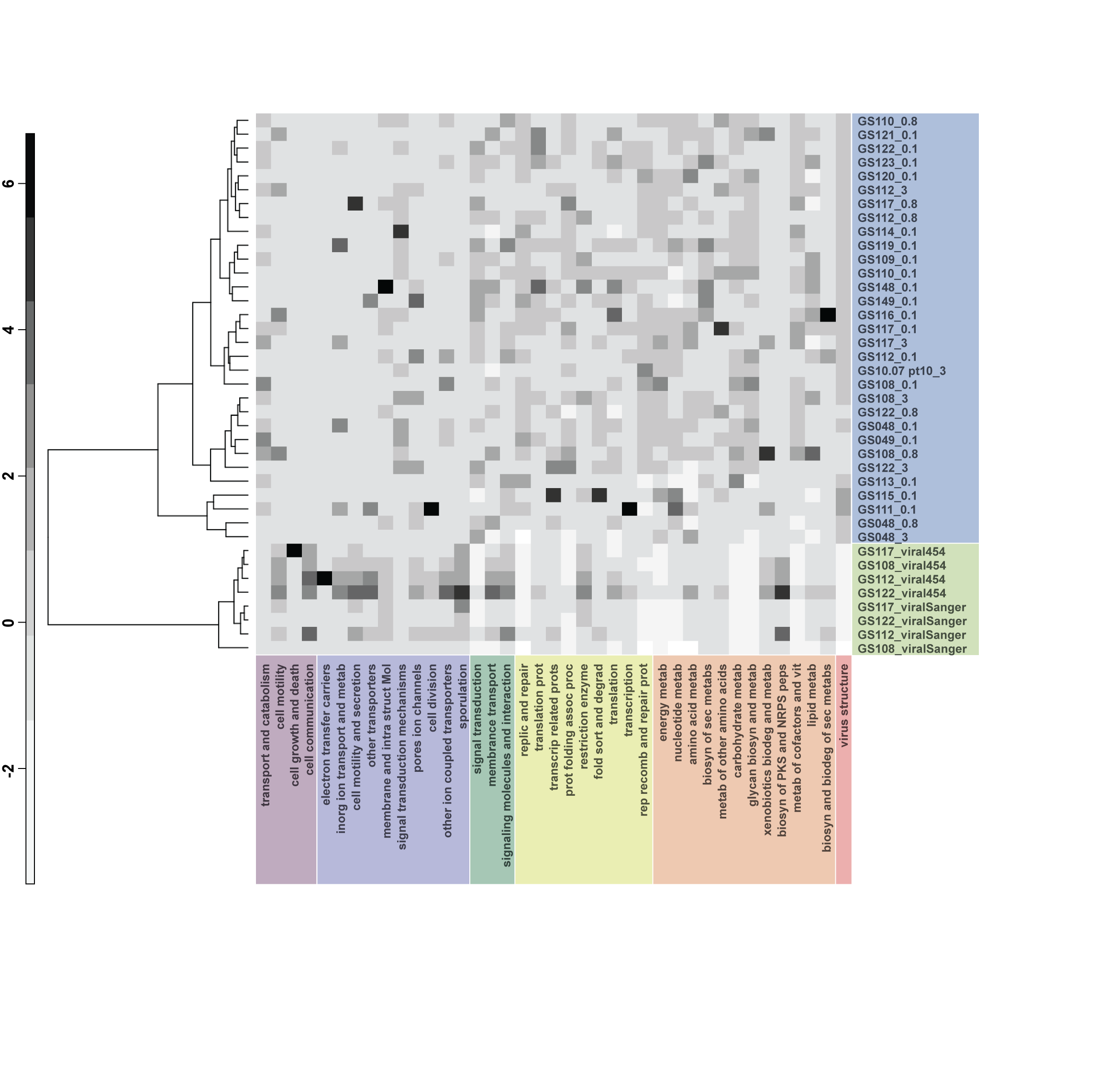

Supplement: Figure S1 — Heatmap showing delineation of viral sequences from large vs. viral fraction. Abundance of sequences from each site within selected functional groups at the level-2 classification of Gene Ontology (GO) was used to generate heatmap. Hierarchical clustering of sites indicates a grouping of large (blue) versus viral (green) fraction. Functional groups are color-coded as follows: cellular processes (purple), cellular processes and signaling (blue), environmental information processing (green), genetic information processing (yellow), metabolism (orange), phage structure (red). (TIF) [file pone.0042047.s002.tif]
